# Renal function had an independent relationship with coronary artery calcification in Chinese elderly men

**DOI:** 10.1186/s12877-017-0470-z

**Published:** 2017-04-07

**Authors:** Shihui Fu, Zhao Zhang, Leiming Luo, Ping Ye

**Affiliations:** 1 Department of Geriatric Cardiology, Chinese People’s Liberation Army General Hospital, Beijing, China; 2 Department of Cardiology and Hainan Branch, Chinese People’s Liberation Army General Hospital, Beijing, China

**Keywords:** Chinese elderly men, Coronary artery calcification, High-definition computerized tomography, Renal function

## Abstract

**Background:**

Although previous studies have analyzed the relationship between renal function and coronary artery calcification (CAC) in pre-dialysis and dialysis patients, limited studies have discussed the relationship between renal function and CAC in Chinese elderly men without obvious damage of renal function. The present study was designed to explore the relationship between renal function and CAC in Chinese elderly men without obvious damage of renal function.

**Methods:**

This cross-sectional study was carried out in 105 male participants older than 60 years with glomerular filtration rate (GFR) ≥ 45 ml/min/1.73 m^2^. CAC was detected by high-definition computerized tomography (HDCT), which is a highly sensitive technique for detecting the CAC and provides the most accurate CAC scores up to date.

**Results:**

Age was 72 ± 8.4 years on average and ranged from 60 to 89 years. Simple correlation analysis indicated that all kinds of CAC scores including the Agatston, volume and mass scores inversely correlated with GFR values (*p* < 0.05 for all). In multivariate linear regression analysis, GFR values were independently associated with all these CAC scores (*p* < 0.05 for all).

**Conclusion:**

Renal function had an independent relationship with CAC detected by HDCT in Chinese elderly men, demonstrating that the relationship between renal function and CAC started at the early stage of renal function decline.

## Background

Coronary artery calcification (CAC) not only is used for the non-invasive diagnosis of coronary artery disease (CAD), but also has the prognostic value for future cardiac events and mortality [1, 2]. Meanwhile, CAC is a common complication of patients suffering from obvious damage of renal function, especially chronic dialysis, and may represent an important mechanism linking the change of renal function with cardiovascular morbidity and mortality [3–6]. Although the available literatures have analyzed the relationship between renal function and CAC in pre-dialysis and dialysis patients, limited studies have discussed the relationship between renal function and CAC in individuals without obvious damage of renal function [7–9]. It is well known that CAC is also identified in individuals without obvious damage of renal function [10, 11]. Additionally, age and gender have an important effect on the relationship between renal function and CAC, and scarce studies have intentionally described this kind of relationship in Chinese elderly men. Given the recent recommendation of glomerular filtration rate (GFR) 45 ml/min/1.73 m^2^ as the cut-off point of renal function status in elderly, only the individuals with GFR ≥ 45 ml/min/1.73 m^2^ were enrolled in the present study, providing an opportunity to be absorbed in the relationship between renal function and CAC in Chinese elderly men without obvious damage of renal function. In the study presented here, CAC was detected by high-definition computerized tomography (HDCT), which is a highly sensitive technique for detecting the CAC and provides the most accurate CAC scores up to date.

## Methods

### Study participants

This cross-sectional study was carried out in 105 male participants hospitalized in Chinese People’s Liberation Army General Hospital. All included were older than 60 years. Exclusion criteria were: 1) participants with previous percutaneous transluminal coronary angioplasty, coronary artery bypass graft, heart valve replacement or cardiac pacemaker implantation; 2) participants with a disorder affecting the bone and calcium metabolism, such as GFR < 45 ml/min/1.73 m^2^, thyrotoxicosis, hyperparathyroidism, neoplasms or infectious diseases; and 3) participants with a drug affecting the bone and calcium metabolism, such as glucocorticosteroid, estrogen or bisphosphonates.

### Clinical data extraction

Detailed history and physical examination were performed on all participants at inclusion into the study. All participants were hospitalized and examined at stable ambient temperature. Height was measured in the standing position using a wall-mounted measuring tape, and body weight was measured with a digital scale wearing a standardized health check-up clothes. Body mass index (BMI) was calculated as weight in kilograms divided by height in meters squared. Mean systolic and diastolic blood pressure (MSBP and MDBP) were measured using a standard mercury sphygmomanometer with participants in a seated position after having quietly rested for 10 minutes. Hypertension was defined as MSBP ≥ 140 mmHg, MDBP ≥ 90 mmHg or receiving the anti-hypertensive drugs. CAD was characterized by the positive diagnostic procedure (stress test, computed tomography, radionuclide image and coronary angiography), and the presence of angina pectoris or myocardial infarction. Participants with atypical angina pectoris or suspected CAD received the examination of stress test in the same room using the same equipment (T-2100 Treadmill, GE Healthcare, Wisconsin, USA), and evaluated by the consensus of two experienced cardiologists. Routine biochemistry was performed on fasting serum sample collected in the morning. Biochemistry values, including triglyceride, low density lipoprotein-cholesterol (LDL-c), high density lipoprotein-cholesterol (HDL-c), fasting blood glucose (FBG), uric acid, calcium and phosphorus, were tested by an automatic analyzer (Cobas® 6000, Roche, Basel, Switzerland). GFR values were determined by Chinese modified Modification of Diet in Renal Disease equation and used for the determination of renal function: 175 × serum creatinine (mg/dl)^-1.234^ × age (year)^-0.179^ × 0.79 (if female) [12].

### Coronary artery calcium scores

**Fig. 1:**
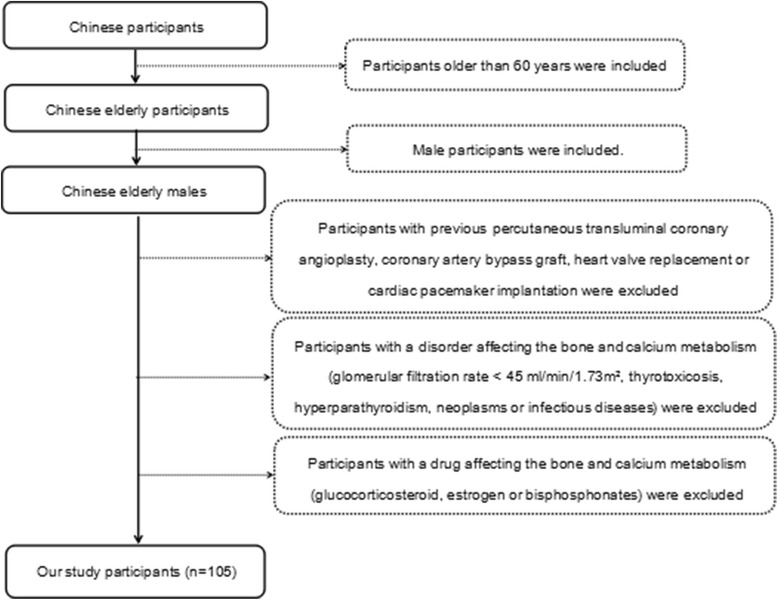
Flow chart of study participants with inclusion and exclusion criteria

Participants underwent the scans of HDCT (Discovery CT 750 HD, GE Healthcare, Wisconsin, USA). All scans were performed in the same room using the same equipment, and analyzed by the consensus of two radiologists with more than two years of experience blinded to all participants and prior information. For defining the quantity of coronary calcium, the Agatston, volume and mass scores were calculated using the software (Smart Score 4.0, GE Healthcare, Wisconsin, USA) on the three-dimensional workstation (Advantage Windows Workstation 4.5, GE Healthcare, Wisconsin, USA), according to the following equations: 1) Agatston score = slice increment/slice thickness × ∑(area × cofactor); 2) volume score = ∑(area × slice increment); and 3) mass score = ∑(area × slice increment × mean CT density) × calibration factor [13, 14]. The sum of all scores for each coronary artery including left main artery, left anterior descending artery, left circumflex artery and right coronary artery was used to generate the total CAC scores (Fig. 1).

### Statistics

Descriptive statistics for the continuous variables with normal distribution were presented as mean ± standard deviation and for the categorical variables were presented as number and percentage. Participants were classified into three groups according to the GFR tertiles as follows: tertile 1 (>86.2 mL/min/1.73 m^2^), tertile 2 (76.2–86.2 mL/min/1.73 m^2^) and tertile 3 (< 76.2 ml/min/1.73 m^2^). Statistical comparison for these groups was conducted using the one-way analysis of variance (ANOVA) for the continuous variables with normal distribution and chi-squared test for the categorical variables. Pearson’s correlation for the continuous variables with normal distribution and Spearman’s correlation for the categorical variables were used to evaluate their simple relationship with CAC scores. Multivariate linear regression analysis (enter) was undertaken after adjusting for age, BMI, CAD, hypertension, MSBP, MDBP, heart rate, triglyceride, LDL-c, HDL-c, FBG, uric acid, calcium and phosphorus values. All analyses were two-sided with *p* value < 0.05 considered statistically significant, and performed using the Statistical Package for the Social Sciences version 17 (SPSS, Inc, Chicago, IL, USA).

## Results

### Baseline characteristics

For the total participants, age was 72 ± 8.4 years on average and ranged from 60 to 89 years. Some participants had the co-morbidities of CAD (67.6%) and hypertension (73.3%), and used the non-steroidal anti-inflammatory drugs (NSAIDs, 61.0%) and angiotensin converting enzyme inhibitors/angiotensin receptor blockers (ACEI/ARBs, 55.2%). No participant used the antibiotics. Baseline characteristics of all participants were compared in Table 1. Compared to those in tertile 1 and 2 of GFR values, participants in tertile 3 were significantly older, more likely to be hypertensive, and had significantly higher all kinds of CAC scores including the Agatston, volume and mass scores (*p* < 0.05 for all). Compared to those in tertile 1 of GFR values, participants in tertile 3 had a significantly greater percentage of CAD (*p* < 0.05). Participants in tertile 2 and 3 of GFR values had significantly higher uric acid values than those in tertile 1 (*p* < 0.05 for all).

**Table 1:** Baseline characteristics of all participants divided by the status of renal function

Characteristics	Overall	Tertile 1	Tertile 2	Tertile 3	*p* value^a^	*p* value^b^	*p* value^c^	*p* value^d^
	(*n* = 105)	(*n* = 35)	(*n* = 35)	(*n* = 35)				
		GFR > 86.2 ml/min/1.73 m^2^	GFR 76.2-86.2 ml/min/1.73 m^2^	GFR < 76.2 ml/min/1.73 m^2^				
Age (year)	72 ± 8.4	70 ± 8.2	70 ± 7.8	76 ± 7.8	0.001	0.940	0.001	0.001
BMI (kg/m^2^)	25 ± 2.7	26 ± 3.1	25 ± 2.3	25 ± 2.8	0.719	0.423	0.769	0.612
CAD (%)	71(67.6)	19(54.3)	23(65.7)	29(82.9)	0.037	0.329	0.010	0.101
Hypertension (%)	77(73.3)	22(62.9)	23(65.7)	32(91.4)	0.012	0.803	0.009	0.004
MSBP (mmHg)	126 ± 15.4	121 ± 15.8	127 ± 14.2	131 ± 14.8	0.021	0.071	0.006	0.340
MDBP (mmHg)	72 ± 10.1	70 ± 10.8	74 ± 9.3	71 ± 10.2	0.294	0.136	0.732	0.249
Heart rate (bpm)	71 ± 10.3	73 ± 12.3	70 ± 8.5	69 ± 9.6	0.275	0.195	0.142	0.862
Triglyceride (mmol/L)	1.5 ± 0.7	1.5 ± 0.6	1.5 ± 0.7	1.6 ± 0.8	0.836	0.895	0.661	0.568
HDL-c (mmol/L)	1.1 ± 0.3	1.1 ± 0.2	1.1 ± 0.3	1.1 ± 0.3	0.740	0.761	0.442	0.642
LDL-c (mmol/L)	2.5 ± 0.8	2.5 ± 0.7	2.6 ± 0.7	2.4 ± 0.8	0.590	0.620	0.595	0.305
FBG (mmol/L)	5.3 ± 0.6	5.5 ± 0.7	5.2 ± 0.5	5.3 ± 0.6	0.132	0.045	0.269	0.360
Calcium (mmol/L)	2.3 ± 0.2	2.3 ± 0.3	2.2 ± 0.1	2.3 ± 0.1	0.240	0.096	0.287	0.545
Phosphorus (mmol/L)	1.1 ± 0.2	1.1 ± 0.1	1.1 ± 0.2	1.1 ± 0.2	0.236	0.422	0.090	0.368
Uric acid (mmol/L)	335.5 ± 74.2	299.6 ± 61.3	342.3 ± 59.9	364.7 ± 85.0	0.001	0.012	<0.001	0.182
GFR (ml/min/1.73 m^2^)	82.0 ± 13.3	96.6 ± 8.3	81.5 ± 2.7	67.9 ± 6.4	<0.001	<0.001	<0.001	<0.001
NSAIDs (%)	64(61.0)	19(54.3)	22(62.9)	23(65.7)	0.594	0.467	0.329	0.803
ACEI/ARB (%)	58(55.2)	18(51.4)	17(48.6)	23(65.7)	0.303	0.811	0.225	0.147
CAC scores								
Agatston score	388.9 ± 739.2	240.4 ± 338.5	248.1 ± 475.6	678.3 ± 1096.1	0.016	0.964	0.012	0.014
Volume score	148.8 ± 263.3	94.8 ± 128.4	102.1 ± 169.8	249.5 ± 388.6	0.020	0.982	0.009	0.010
Mass score	50.7 ± 92.7	31.5 ± 45.0	32.0 ± 64.0	88.6 ± 134.0	0.011	0.906	0.013	0.018

^a^Total; ^b^Tertile 2 vs. Tertile 1; ^c^Tertile 3 vs. Tertile 1; ^d^Tertile 3 vs. Tertile 2

*GFR* Glomerular filtration rate, *BMI* Body mass index, *CAD* Coronary artery disease, *MSBP* Mean systolic blood pressure, *MDBP* Mean diastolic blood pressure, *HDL-c* High density lipoprotein-cholesterol, *LDL-c* Low density lipoprotein-cholesterol, *FBG* Fasting blood glucose, *NSAIDs* Non-steroidal anti-inflammatory drugs, *ACEI/ARBs* Angiotensin converting enzyme inhibitors/angiotensin receptor blockers, *CAC* Coronary artery calcification

### Association between GFR values and CAC scores

As shown in Table 2, simple correlation analysis indicated that all kinds of CAC scores including the Agatston, volume and mass scores positively correlated with age and CAD, and inversely correlated with GFR and LDL-c values (*p* < 0.05 for all). In multivariate linear regression analysis (Table 3), CAD was independently associated with all kinds of CAC scores including the Agatston, volume and mass scores, and GFR values were independently associated with all these CAC scores (*p* < 0.05 for all).

**Table 2:** Relationship of coronary artery calcium scores with renal function and other variables in simple correlation analysis

Characteristics	Agatston score		Volume score		Mass score	
	*r* value	*p* value	*r* value	*p* value	*r* value	*p* value
Age (year)	0.258	0.008	0.247	0.011	0.267	0.006
BMI (kg/m^2^)	0.117	0.236	0.129	0.188	0.089	0.369
CAD (%)	0.260	0.007	0.254	0.009	0.253	0.009
Hypertension (%)	0.182	0.062	0.196	0.045	0.193	0.048
MSBP (mmHg)	0.182	0.064	0.205	0.036	0.182	0.064
MDBP (mmHg)	0.054	0.582	0.078	0.430	0.044	0.655
Heart rate (bpm)	−0.165	0.093	0.169	0.084	−0.177	0.070
Triglyceride (mmol/L)	−0.034	0.727	−0.041	0.680	−0.049	0.616
HDL-c (mmol/L)	−0.030	0.762	−0.045	0.651	−0.031	0.757
LDL-c (mmol/L)	−0.206	0.035	−0.206	0.035	−0.226	0.020
FBG (mmol/L)	0.035	0.725	0.052	0.599	0.047	0.633
Calcium (mmol/L)	−0.093	0.346	−0.080	0.414	−0.102	0.302
Phosphorus (mmol/L)	0.129	0.191	0.121	0.221	0.113	0.251
Uric acid (mmol/L)	0.041	0.678	0.026	0.794	0.032	0.750
GFR (ml/min/1.73 m^2^)	−0.251	0.010	−0.246	0.011	−0.255	0.009

*BMI* Body mass index, *CAD* Coronary artery disease, *MSBP* Mean systolic blood pressure, *MDBP* Mean diastolic blood pressure, *HDL-c* High density lipoprotein-cholesterol, *LDL-c* Low density lipoprotein-cholesterol, *FBG* Fasting blood glucose, *GFR* Glomerular filtration rate

**Table 3:** Relationship of coronary artery calcium scores with renal function and other variables in multivariate linear regression analysis

Characteristics	Agatston score		Volume score		Mass score	
	β value	*p* value	β value	*p* value	β value	*p* value
Age (year)	0.115	0.344	0.104	0.390	0.109	0.366
BMI (kg/m^2^)	0.150	0.193	0.151	0.189	0.116	0.312
CAD (%)	0.209	0.048	0.206	0.050	0.215	0.040
Hypertension (%)	0.002	0.985	0.008	0.944	0.003	0.978
MSBP (mmHg)	−0.007	0.958	0.006	0.962	−0.008	0.953
MDBP (mmHg)	0.010	0.935	0.032	0.795	0.013	0.917
Heart rate (bpm)	−0.139	0.167	−0.138	0.170	−0.154	0.125
Triglyceride (mmol/L)	0.038	0.728	0.021	0.845	0.033	0.758
HDL-c (mmol/L)	0.096	0.383	0.080	0.467	0.085	0.440
LDL-c (mmol/L)	−0.153	0.160	−0.157	0.147	−0.164	0.129
FBG (mmol/L)	0.040	0.693	0.054	0.590	0.060	0.555
Calcium (mmol/L)	−0.063	0.542	−0.040	0.701	−0.064	0.536
Phosphorus (mmol/L)	0.108	0.342	0.094	0.405	0.098	0.385
Uric acid (mmol/L)	−0.099	0.414	−0.106	0.382	−0.099	0.413
GFR (ml/min/1.73 m^2^)	−0.230	0.044	−0.228	0.045	−0.232	0.042

*BMI* Body mass index, *CAD* Coronary artery disease, *MSBP* Mean systolic blood pressure, *MDBP* mean diastolic blood pressure, *HDL-c* High density lipoprotein-cholesterol, *LDL-c* Low density lipoprotein-cholesterol, *FBG* Fasting blood glucose, *GFR* Glomerular filtration rate

## Discussion

HDCT is a highly sensitive technique for detecting the CAC and provides the most accurate CAC scores up to date. The study presented here advocated that renal function had an independent relationship with CAC detected by HDCT in elderly men with GFR ≥ 45 ml/min/1.73 m^2^. Patients with obvious damage of renal function appear to have an increased risk of CAD [15–17]. One of the possible underlying mechanisms is that the decline of renal function leads to the acceleration of CAC [18]. CAC is not only applied for the non-invasive diagnosis of CAD, but also a significant predictor of future cardiovascular morbidity and mortality [1, 2]. Meanwhile, CAC is frequent in patients suffering from obvious damage of renal function, especially chronic dialysis, and contributes to their increased cardiovascular morbidity and mortality [3]. Previous studies have shown that obvious damage of renal function is apparently related to CAC in pre-dialysis and dialysis patients, but these studies are comprised of relatively young individuals with GFR values in very low range [6–8].

CAC is highly prevalent even in individuals without obvious damage of renal function. In spite of the relationship between renal function and CAC observed in patients with obvious decline of renal function, there is an undefined one between renal function and CAC in individuals without obvious damage of renal function [10, 11, 19]. Moreover, age and gender have an important effect on this kind of relationship, and scarce studies have confirmed that renal function is relevant to CAC in Chinese elderly men. Due to the recent recommendation of GFR 45 ml/min/1.73 m^2^ as the cut-off value of renal function status in elderly, only the individuals with GFR ≥ 45 ml/min/1.73 m^2^ were involved in the present study, providing an opportunity to be absorbed in the relationship between renal function and CAC in Chinese elderly men without obvious damage of renal function. In the present study, the relationship between renal function and CAC started early in the course of renal function decline among Chinese elderly men. Coronary calcification at the early stage of renal function decline may represent the intimal arterial sclerosis and medial vessel calcification.

## Conclusion

In the study presented here, renal function had an independent relationship with CAC detected by HDCT in Chinese elderly men, demonstrating that the relationship between renal function and CAC started at the early stage of renal function decline.

## Abbreviations

CAC: Coronary artery calcification
GFR: Glomerular filtration rate
HDCT: High-definition computerized tomography
